# Quality assessment of heatstroke videos on TikTok

**DOI:** 10.3389/fpubh.2024.1446003

**Published:** 2024-09-04

**Authors:** Jun Qiu, You-Lian Zhou

**Affiliations:** Department of Critical Care Medicine, Chengdu First People's Hospital, Chengdu, Sichuan, China

**Keywords:** heatstroke, TikTok, DISCERN, information quality, social media

## Abstract

**Background:**

The prevalence of heatstroke is rising due to global warming, making it a serious but preventable condition, highlighting the urgent need for effective dissemination of relevant health education to the general public. Advances in technology have made accessing health information more convenient and rapid. In recent years, short videos have become a primary medium for delivering health education, with TikTok gaining considerable popularity among the general public. However, the quality of heatstroke-related health education content on TikTok deserves closer scrutiny.

**Objective:**

This study aimed to evaluate the quality and content of heatstroke-related videos available on TikTok.

**Methods:**

The present study analyzed the top 100 heatstroke-related short videos on TikTok, focusing on their characteristics, quality, and the content they conveyed. The quality of these videos was assessed using the DISCERN instrument. In addition, the completeness of the videos was assessed by examining six key aspects: disease definition, clinical manifestations, risk factors, assessment, management, and outcomes.

**Results:**

The study included a total of 90 videos. The results showed that news organizations and healthcare professionals were the primary contributors to these videos, with those from news organizations receiving the most attention. In contrast, those from healthcare professionals received comparatively less engagement. Overall, the quality of the information was found to be moderately low, with the highest quality videos posted by non-profit organizations, followed by those posted by healthcare professionals. The majority of videos uploaded described the disease definition, clinical presentation, risk factors, assessment, management, and outcomes of heatstroke.

**Conclusion:**

The quality of information provided in heatstroke-related short videos on TikTok is generally inadequate and requires significant improvement. In addition, such content should be subject to government review to ensure its accuracy and reliability.

## Introduction

With global warming, the prevalence of heatstroke is increasing every year. The incidence rate of heatstroke among hospitalized patients in the United States is 36.3 cases per 100,000 people, with an overall mortality rate of 5%. In addition, previous studies have shown that the mortality rate in the intensive care unit (ICU) is significantly higher, ~26.5% for exertional heatstroke (EHS) and 63.2% for classic heatstroke (CHS) (1). It has been reported that the average cost of hospitalization for heatstroke is $17,372, representing a significant economic and disease burden (2). Heatstroke is divided into two distinct categories: classic heat stroke (CHS) and exertional heat stroke (EHS) (1). It is characterized by an elevated core body temperature above 40°C, accompanied by central nervous system dysfunction and multiple organ dysfunction (1, 2). Clinical manifestations of heatstroke include high fever, delirium, tachycardia, and dry skin, among others. In addition, complications such as renal failure, coagulopathy, and respiratory failure may occur (2). Because there are many ways to prevent heatstroke before it is diagnosed, there is an urgent need to educate the public about the prevention and dangers of heatstroke.

More and more people are concerned about their health and often seek health information online (e.g., WeChat, Baidu, various health apps, short videos, etc.) before consulting a professional doctor. According to the 50th Statistical Report on China's Internet Development Status released by the China Internet Network Information Center, as of June 2022, China's Internet penetration rate was 74.45%, with 1.051 billion Internet users (3–5). In recent years, the unprecedented increase in short-form video applications has changed the way people obtain health information. The number of short-form video users has increased significantly, with ~962 million users reported nationwide (3). These video platforms provide individuals with greater access to health education information online (6, 7). Short-form video platforms such as TikTok have emerged as new channels for the public to receive health education information (8, 9). We chose TikTok for this study because it is the fastest-growing short-form video application and is very popular among the general public, especially among 13–24-year-olds (10, 11).

In alignment with the findings of recent research, the restrictions imposed by the pandemic resulted in a notable decline in travel and a concomitant increase in concern about personal health. This resulted in a notable increase in the utilization of digital resources offering health information, including short videos and applications. Notably, videos on TikTok about the novel coronavirus received over 93 million views (12, 13). However, TikTok, which was launched in 2016, is a relatively nascent social media platform; thus, there is a lack of comprehensive studies examining the quality of its content. A number of studies have demonstrated that ~½ of the videos on TikTok disseminate misinformation or low-quality knowledge (14, 15). Some studies have revealed the dissemination of misinformation about both the novel coronavirus and prostate cancer on the social media platform TikTok, indicating that the platform is unable to provide satisfactory healthcare information (16–18). A prior investigation also revealed that the quality of TikTok videos about gallstones was suboptimal, prompting the researcher to conclude that TikTok was an inadequate conduit for disseminating health-related information (19). In light of these concerns, disseminating misinformation on TikTok has been a topic of significant interest. The extent to which TikTok affects healthcare communication remains uncertain.

A review of the available data on TikTok indicates a proliferation of content related to heatstroke. Nevertheless, the veracity and quality of the information remain undetermined. At the time of writing of this article, no published articles have assessed the quality of the information shared on TikTok regarding heatstroke-related videos. The objective of this study is to evaluate the reliability and accuracy of videos about heatstroke on TikTok and to establish a foundation for a more comprehensive understanding of the role that TikTok plays in disseminating healthcare information.

## Methods

### Retrieval strategy and data collection

On 5 October 2022, we conducted a comprehensive search of TikTok videos using the Chinese keyword “热射病.” TikTok's search function offers three sorting criteria: “Comprehensive,” “Latest Release,” and “Most Likes,” with “Comprehensive” being the default. We used the default sort to retrieve the top 100 videos about heatstroke because that is what the majority of users rely on. Our selection of the top 100 videos is based on previous research, which clearly shows that over 95% of users only watch the first 60 videos (16). Each video was reviewed individually, and 90 videos were finally included in the study based on the inclusion and exclusion criteria. For each video, we documented the following characteristics: uniform resource locator (URL), upload date, account identity information, account number of fans, account likes, video likes, video comments, video collection, and length.

### Inclusion and exclusion criteria

**Figure 1:**
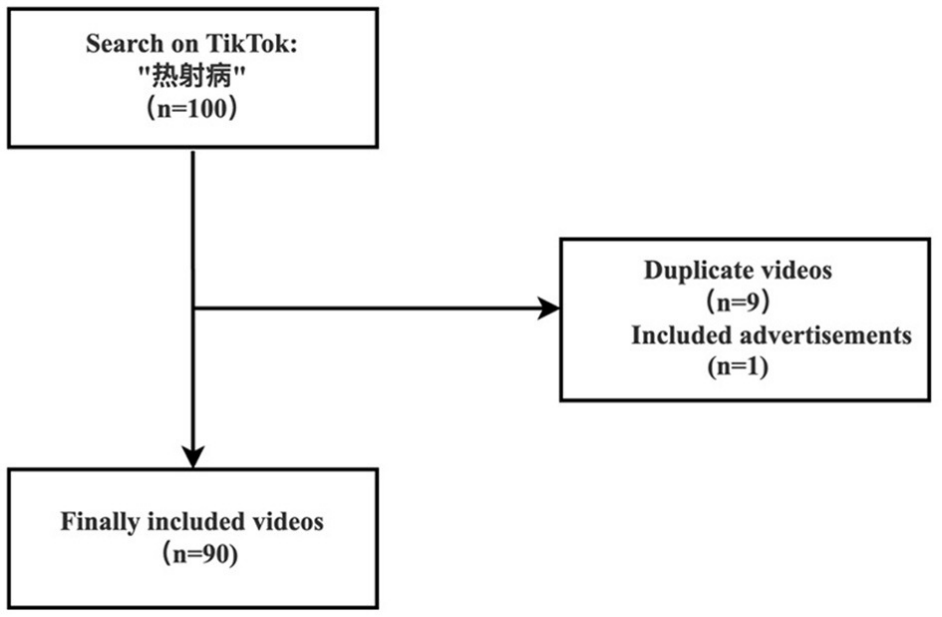
Video screening flowchart.

The inclusion criteria were videos that provided health information about heatstroke. The exclusion criteria were as follows: (1) duplicate videos and (2) videos that contained advertisements. A detailed description of the specific flow chart is provided in Figure 1.

### Measures

The evaluation of the TikTok short videos was carried out from two different perspectives: the quality of the video information and the content of the video.

### Information quality

Mobile health (mHealth) uses mobile phones to educate and improve access to health information. Various evaluation tools are used to assess the effectiveness of mHealth applications. For example, Moumane et al. have used the Confidence in Online Shopping Measurement Index for Consumers (COSMIC) functional scale to assess these applications (20). Similarly, other studies have used the DISCEN scoring tool. These tools help quantify the functionality and utility of mobile health services to ensure that they meet the required education and healthcare delivery standards. We used the DISCERN scoring tool to assess the quality of the information. DISCERN was originally developed to assess the quality of written health information about treatment options (21). More recently, it has been used to assess the information quality of health education content in videos (22). Several studies have used this tool to evaluate the educational quality of TikTok videos on diabetes, chronic obstructive pulmonary disease (COPD), urinary tract tumors, and other related conditions (23–26). In addition, international researchers have used it to evaluate the information quality of tumor-related videos on YouTube (22). The DISCERN instrument comprises three parts with a total of 16 items: the first part assesses the credibility of the video (eight items), the second part assesses the quality of the treatment decisions (seven items), and the last part assesses the overall quality (one item). Each item is rated on a scale from 1 (lowest) to 5 (highest) (21).

### Video content

Video content was evaluated using Goobie et al.'s six categories: disease definition, signs and symptoms, risk factors, assessment, management, and outcomes (27). Each item was evaluated on a scale of 0–2 points, with the following criteria: no content (0 points), some content (1 point), and extensive content (2 points).

### Assessment process

All videos were independently reviewed by two investigators (JQ and YL Z), both intensivists. Continuous variables were presented as either mean and standard deviation or median and interquartile range. Categorical variables were presented numerically with the corresponding percentage. Data were compiled using Excel 2022, and statistical analysis was performed using SPSS 26.0.

## Results

### Video characteristics

A classification of video sources based on account information reveals two categories: individual users and organizational users. Individual users can be further divided into healthcare professionals and science communicators. Organizational users can be further divided into three categories: news organizations, non-profit organizations, and for-profit organizations. The results showed that healthcare professionals uploaded the most videos, with a total of 27 videos representing 30% of the total sample. Next, science communicators uploaded 11 videos, representing 12.2% of the total sample. Among organizational users, news organizations uploaded the most videos related to heatstroke, with a total of 39 videos representing 43.3% of the total sample. This was followed by non-profit organizations with 11 videos (12.2%) and for-profit organizations with two videos (2.2%; Table 1).

**Table 1:** Descriptions of video sources.

**Source**	**Description**	**Video (%)**
Science communicators	Individuals who specialize in translating complex scientific ideas into accessible formats	11 (12.2%)
Health professionals	Individuals who have received specialized education in healthcare [e.g., doctors, nurses]	27 (30%)
News organizations	Organizations whose main purpose is to gather and distribute news and information [TV account, newspaper]	39 (43.3%)
For-profit organizations	Companies that operate to generate income	2 (2.2%)
Non-profit organizations	Organizations that exist to serve a specific charitable, educational purpose [e.g., hospitals, colleges]	11 (12.2%)

The present study included a total of 90 short videos related to heatstroke. The most recent video in the sample was uploaded 42 days prior to data collection, while the oldest was uploaded 500 days prior. There was a considerable range in the duration of the videos, with the longest video being 275 s and the shortest being 0.5 s. The average video duration was 90 s. Among the video accounts, news organizations had the largest number of followers, i.e., 4.225 million, followed closely by science communicators, 4.205 million.

In contrast, the least popular accounts were those belonging to non-profit organizations, with only 0.56 million followers. In total, the videos received 8.8 million likes and 1.1 million comments. The most popular videos posted by news organizations received 1.23 million likes. Notably, videos from science communicators received the most comments, likes, and collections, while those from non-profit organizations received the fewest (Table 2).

**Table 2:** Analysis of video characteristics.

**Source type**	**Account fans**	**Account likes**	**Days on TikTok**	**Length**	**Video comments**	**Video likes**	**Collection**
Health professionals (Median)	2,133,000	11,838,000	82	108	109	3486	192
IQR	(590,000, 2,777,000)	(3,031,000, 21,863,000)	(78, 83)	(60, 139)	(64, 1,469)	(1,241,43,000)	(100, 1,475)
Science communicators (Median)	4205000	98,367,000	83	111	5252	86000	11000
IQR	(2973000,19484000)	(49,080,000, 440,000,000)	(83, 84)	(76, 174)	(1,135, 23,000)	(25,000, 220,000)	(1,666, 30,000)
News organization (Median)	4,255,000	90,903,000	83	53	1629	18000	2302
IQR	(2,061,000, 10,625,000)	(16,509,000, 360,000,000)	(81, 84)	(16, 120)	(219, 11,000)	(3,601, 75,000)	(223, 5,701)
Non-profit organizations (Median)	569,000	3,561,000	83	95	68	623	80
IQR	(252,000, 1,186,000)	(1,731,000, 5,126,000)	(81, 83)	(48, 120)	(36, 185)	(407, 1,908)	(43, 259)
For-profit organizations (Median)^*^	2,840,000	49,628,000	82		342	2410	341
IQR	-	-	-		-	-	-

^*^Quartile distance could not be calculated due to insufficient data.

The quality of the information presented in the short videos was evaluated using the DISCERN instrument, which assesses the reliability of the information, the quality of the information in terms of treatment choice, and the overall quality of the information. The overall score was less than satisfactory. The reliability scores for the short videos ranged from 10.5 to 18.4, with a total possible score of 40. The quality of the treatment choices ranged from 7 to 11.8, with a total possible score of 35. Overall information quality scores ranged from 1.5 to 2.7, with a total possible score of 5. Specifically, non-profit organizations had the highest reliability, followed by healthcare professionals, while for-profit organizations had the lowest reliability. In terms of quality of treatment choice, videos from healthcare professionals were rated the highest, followed by those from non-profit organizations, with videos from for-profit organizations being rated the lowest. In terms of overall video quality, our results showed that videos uploaded by healthcare professionals received the highest scores, followed by videos from non-profit organizations and news organizations, with videos from for-profit organizations receiving the lowest scores (Table 3).

**Table 3:** DISCERN scores of heatstroke videos on TikTok.

	**Video reliability**	**Quality of treatment choice**	**Overall quality**	**Total score**
**Healthcare professionals**				
Mean (SD)	15.1 (3.1)	11.8 (3.2)	2.7 (0.9)	29.6 (5.5)
Median	15	11	3	29
**Science communicators**				
Mean (SD)	12.1 (3.6)	8.5 (2.2)	1.6 (0.7)	22.1 (4.8)
Median	12	7	1	21
**News organizations**				
Mean (SD)	12.7 (3.0)	8.8 (2.4)	1.9 (0.9)	23.4 (4.9)
Median	12	8	2	21
**Non-profit-organizations**				
Mean (SD)	18.4 (5.2)	11.1 (3.7)	2.5 (1.1)	31.9 (7.5)
Median	16	10	3	29
**For-profit organizations**				
Mean (SD)	10.5 (2.1)	7.0 (0)	1.5 (0.7)	19.0 (1.4)
Median	11	7	1.5	19

### Short video content

The short videos on heatstroke evaluated in this study cover six essential aspects: disease definition, clinical manifestations, risk factors, assessment, management, and outcomes. Our results showed that the majority of videos (*n* = 43, 47.8%) clearly explained the definition of heatstroke, while a small number (*n* = 10, 11.1%) had no content on this aspect. More than half of the videos had some content on clinical manifestations (*n* = 69, 76.7%), risk factors (*n* = 77, 85.6%), assessment (*n* = 48, 53.3%), management (*n* = 68, 75.6%), and outcomes (*n* = 78, 86.7%; Table 4).

**Table 4:** Completeness of video content.

**Content**	**No content**	**Some content**	**Extensive content**
Definition [*N*, %]	10 (11.1)	37 (41.1)	43 (47.8)
Clinical manifestations [*N*, %]	5 (5.6)	69 (76.7)	16 (17.8)
Risk factors [*N*, %]	4 (4.4)	77 (85.6)	9 (10.0)
Evaluation [*N*, %]	40 (44.4)	48 (53.3)	2 (2.2)
Management [*N*, %]	15 (16.7)	68 (75.6)	7 (7.8)
Outcomes [*N*, %]	11 (12.2)	78 (86.7)	1 (1.1)

## Discussion

The recent proliferation of short video applications, including TikTok, Bili Bili, and YouTube, has been remarkable. TikTok is a particularly prominent platform in the digital landscape. Its exponential growth can largely be attributed to several factors, including frequent updates, diverse content, short video formats, and recommendations based on personal preferences (28). The full extent of the impact of heatstroke messages delivered through digital platforms has yet to be determined through comprehensive research. In particular, the impact of messages delivered in heatstroke videos on the social media platform TikTok remains unclear. This study includes 90 heatstroke videos on TikTok, with the selected videos receiving a total of 8.8 million likes and 1.1 million comments. The results of this study suggest that the social media platform TikTok may be an effective channel for disseminating information related to heatstroke.

Our analysis suggests that the overall quality of information about heatstroke in TikTok videos falls below acceptable standards. This is consistent with the findings of Meade and Dreyer, who highlighted the poor quality of orthodontic videos on TikTok. Similarly, Paulina Sledzinska et al. analyzed the meningioma content on YouTube using the DISCERN tool and reported an average score of 36.4, indicating poor quality. They also found that videos about Takotsubo syndrome had an average score of 36.93, indicating poor quality 2 (22, 24, 29). In contrast to our findings, however, the study by Kong et al. found that the overall DISCERN score for short videos about diabetes on TikTok ranged from 40 to 50. In addition, the researchers indicated that the quality of these videos was considered acceptable on average (25). In the same year, Song et al. published another article evaluating the information quality of short COPD videos on TikTok. The total video DISCERN scores ranged from 56 to 66.8, leading them to conclude that the overall information quality of COPD videos on TikTok was satisfactory (23).

Nevertheless, the overall DISCERN scores of the videos in our study ranged from 19 to 31.9, a range that was significantly lower than that observed in previous studies. This phenomenon can be attributed to several factors. First, there is increased awareness of chronic diseases such as chronic obstructive pulmonary disease (COPD) and diabetes mellitus (DM) due to their high prevalence. In contrast, heatstroke is a critical illness with a relatively low incidence, and its attention in China has only begun in recent years. Second, heatstroke is a type of heat exhaustion, which is a relatively unfamiliar concept to the general public. Even among healthcare professionals and non-profit organizations, the survey results indicate that their understanding is still inadequate.

Video sources can be classified into two categories: individual users and organizational users. Subsequently, the categories were further subdivided into five subcategories. The findings indicated that news organizations uploaded the greatest number of videos, followed by healthcare professionals, and for-profit organizations uploaded the fewest videos. In terms of popularity, news organizations have the greatest number of followers and the second-highest number of comments, likes, and favorites for the short videos they produce. However, a potential issue is the finding that the quality of the information available only ranked third. A total of one-third of the videos were uploaded by healthcare professionals, and the quality of the information presented in these videos was also relatively high. This finding is consistent with previous reports in the literature indicating that videos produced by professionals are more likely to be of higher quality (30, 31). However, the accounts exhibited a markedly low number of followers and received only a small number of comments, likes, and favorites. News organizations are adept at disseminating information, and the information they disseminate is more compelling. Despite their high level of medical expertise, healthcare professionals often exhibit shortcomings in their capacity to disseminate information in an efficacious manner. Future development of TikTok may involve combining these two resources to promote medical knowledge and health information in a more effective manner.

In terms of video content, the vast majority of videos presented a definition of heatstroke, with 47.8% offering comprehensive content and 41.1% providing partial content. Concurrently, over half of the videos address the symptoms, risks, assessment, management, and outcomes of heat stroke. It can thus be concluded that the dissemination of short videos is relatively comprehensive in terms of heatstroke content, covering all aspects of the disease. The findings of the study by Xu et al. indicated that the general community population demonstrated a satisfactory level of knowledge, positive attitudes, and appropriate practices regarding heatstroke. Furthermore, as indicated in the article, the majority of participants displayed a favorable disposition toward acquiring knowledge and disseminating it to others (32). This finding indicates that the public has a general understanding of the fundamentals of heatstroke, which may be attributed to the diverse methods of popularizing science, particularly the Internet.

This study has several limitations. First, it should be noted that the study in question only considered the quality of content related to heatstroke on the social media platform TikTok and did not consider other social media platforms. Considering the aforementioned limitations, the findings of the study may not be applicable to a broader range of social media platforms. Second, due to the rapid update cycle of short videos, this study only included content from a single point in time, which may limit the reproducibility of the results. Third, the study employed the DISCERN instrument to evaluate the quality of the information presented in the videos. Nevertheless, several alternative assessment tools are available for consideration, including the Patient Education Materials Assessment Tool for Audiovisual Materials (PEMAT-A/V), the Journal of American Medical Association (JAMA) benchmark, the Health on the Net (HON) guidelines, and Mobile App Rating Scale (MARS). As a result, the findings may be inherently biased and lack sufficient representativeness.

## Conclusion

We conducted a study of heatstroke-related videos on TikTok, the most popular short-form video app. We found that news organizations and healthcare professionals uploaded the most videos. While news organizations received a high number of followers, comments, likes, and favorites, videos from healthcare professionals were less popular. In terms of video quality, although non-profit and healthcare professional videos ranked first and second, the overall quality was moderately low. Therefore, there is a need for professionals and relevant authorities to monitor the quality of health-related short videos to help the public with early prevention and treatment of diseases, thereby reducing both economic and health burdens.

## Data Availability

The raw data supporting the conclusions of this article will be made available by the authors, without undue reservation.
